# Chitin, Chitinase Responses, and Invasive Fungal Infections

**DOI:** 10.1155/2012/920459

**Published:** 2011-12-11

**Authors:** Karina Vega, Markus Kalkum

**Affiliations:** Department of Immunology and Irell & Manella Graduate School of Biological Sciences, Beckman Research Institute, City of Hope, 1500 East Duarte Road, Duarte, CA 91010, USA

## Abstract

The human immune system is capable of recognizing and degrading chitin, an important cell wall component of pathogenic fungi. In the context of host-immune responses to fungal infections, herein we review the particular contributions and interplay of fungus and chitin recognition, and chitin-degrading enzymes, known as chitinases. The mechanisms of host chitinase responses may have implications for diagnostic assays as well as novel therapeutic approaches for patients that are at risk of contracting fatal fungal infections.

## 1. Introduction

Recipients of solid organs and hematopoietic cell transplants, AIDS patients, and burn victims are usually immunosuppressed for extended periods of time. Their prolonged immunosuppressive state is associated with a high risk of contracting invasive fungal infections (IFIs) [1, 2]. Most IFIs advance rapidly and are often not diagnosed early enough for antifungal drugs to function with full efficacy; therefore, the majority of these infections lead to death [1].

In contrast to immunosuppressed patients, immunocompetent individuals are protected from fungal infections by their functional innate immune system, which readily recognizes and eliminates fungal invaders. Recognition of fungal cellular features by the immune system appears to be a key component of the human antifungal defense [3]. For example, *β*-glucan on the fungal cell wall is recognized as a pathogen-associated molecular pattern (PAMP) by dectin-1 and activates pro- and anti-inflammatory cytokines in a myeloid-differentiation-primary-response-gene-88-(MYD88-) dependent signaling pathway [3–6]. An important component of the fungal cell wall that has not been fully explored as a PAMP is chitin, a polymer of *N*-acetylglucosamine [3, 7]. Chitin is one of the most abundant biopolymers, probably almost as abundant as cellulose [8, 9] and is found on fungal cell walls and exoskeletons of numerous organisms including parasitic worms (helminths) and arthropods. Although humans do not biosynthesize chitin, they do express chitin degrading enzymes, known as chitinases [10–12]. There are two known human chitinases that have chitinolytic activity, chitotriosidase (CHIT-1) and acidic mammalian chitinase (AMCase), as well as multiple noncatalytically active chitinases called chi-lectins [11–14]. The functions of CHIT-1 and AMCase are unknown, but they are thought to aid in the defense of chitin-containing pathogens. For instance, in guinea pigs, serum chitotriosidase levels increase in response to systemic fungal infection [15]. That chitinase levels can vary in response to fungal infections suggests the possibility of using host chitinase responses as a diagnostic. However, several other stimuli can also upregulate chitinase activity [16–19] and counterproductively, several polymorphisms in the *CHIT-1* and *AMCase* genes are known to decrease chitinase activity [20–24]. Thus, there are several challenges to be overcome if chitinase responses were to be used in the diagnosis of fungal infections. More recently, recombinant CHIT-1 was shown to have antifungal properties both *in vitro* and *in vivo*, suggesting the possibility of a gene therapy approach [25]. This paper will explore chitinase responses to fungal infections, current knowledge about the mechanism of chitin recognition by host-immune cells, and regulation of host-chitinase induction.

## 2. Invasive Fungal Infections (IFIs)

Fungal infections have become a major disease concern over the last three decades, in particular for recipients of solid organs and hematopoietic stem cells, AIDS patients, and burn victims, all of whom are usually immunosuppressed for extended periods of time [26–28]. Their prolonged immunosuppressed status leads to an increased risk of contracting opportunistic IFIs. IFIs are also on the rise in intensive care settings, likely due to a growing use of procedures with invasive medical devices and long-term use of antibiotics [29]. In all cases, the most common etiological agents are *Candida albicans* and *Aspergillus fumigatus* [27, 29].

Humans are exposed to hundreds of fungal spores each day, usually without a negative effect on their health. In the lungs of patients that lack sufficient pulmonary immune defenses, *A. fumigatus* fungal spores are able to swell, germinate, and branch into fungal hyphae. The infection can then disseminate to other organs through the bloodstream [26, 30]. Healthy individuals are able to eliminate fungal spores by mucociliary clearance, macrophages, and other primarily pulmonary defense mechanisms [26]. *C. albicans,* on the other hand, is a commensal organism residing in the gastrointestinal tract and oral, and vaginal mucosa of most healthy individuals, where it typically does not produce harmful side effects. However, *Candida* overgrowth can become symptomatic causing mucosal membrane infections, the most common being thrush and vaginal *candidiasis *[31–33]. Severe systemic *Candida *infections (*Candidemia)* and dissemination to internal organs can occur in immunocompromised patients [31–33].

Current methods for detecting IFIs are based on clinical signs and microbial examination. For example, pulmonary fungal infections are typically examined via CT scan, followed up with bronchoalveolar lavage (BAL) and biopsy [27, 30]. Systemic yeast infections such as candidiasis can be diagnosed by the blood culture [33]. However, current diagnostic methods usually do not detect fungal infections at early stages, and therefore, antifungal drug treatment is oftentimes inefficient or delayed. There are some serological tests that may be routinely performed assisting in the diagnosis of fungal infections via detection of fungal antigens in suspected patients [30]. For example, the galactomannan assay is sometimes used for the detection of *Aspergillus* in serum and BAL fluid. This assay works by detecting galactomannan released from the fungal pathogen by enzyme-linked immunosorbent assays [34, 35]. Elevated levels of galactomannan have been detected at early stages of fungal infections, however, the sensitivity and specificity of this assay has been criticized [36]. Moreover, the galactomannan assay is not useful for other fungal pathogens, including *Candida* [30, 37]. *β*-1,3-glucan serological detection assays are more widely used today because they can detect a wide range of fungi, including *Aspergillus* and *Candida, *but they do not detect zygo- or mucormycosis or cryptococcal disease [38]. The *β*-glucan assay works by detecting *β*-1,3-glucan, a major component of the fungal cell wall, circulating in the patient bloodstream [39, 40]. The assay has had great promise for fungal detection, especially when used to confirm galactomannan positive results, however, problems with false-positive (and false-negative) results have been reported [41, 42]. Despite the availability of such diagnostic tests, Garcia-Vidal et al. reported an increase in IFIs and lack of detection at an early stage, within 40 days after hematopoietic cell transplant in infected patients, demonstrating the ineffectiveness of present day diagnostic methods [27].

## 3. Fungal Cell Wall Components and Pattern Recognition Receptors

**Figure 1:**
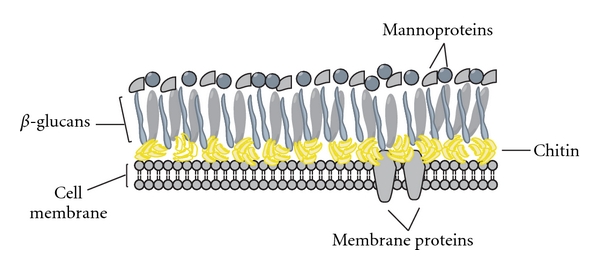
Fungal cell wall components. The fungal cell wall contains a cell membrane with various membrane proteins, a protective layer of chitin (yellow) as well as glucans (mostly beta), and mannoproteins on its surface. Different fungal cell walls contain different glucans. For example, the cell wall of *A. fumigatus* contains *β*-1,3- and *β*-1,4-glucan, and *α*-1,3-glucan [30], while *C. albicans* contains *β*-1,3- and *β*-1,6-glucan [44].

**Figure 2:**
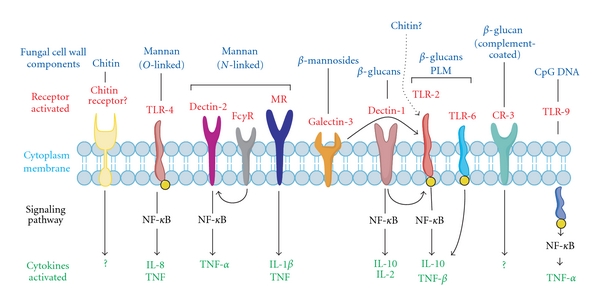
Fungal cell wall pathogen-associated molecular patterns (PAMPs) and their host-pattern recognition receptors (PRRs). Various fungal cell wall components are recognized by specific PRRs. Some PAMPs are recognized by multiple PRRs; for example, *N*-linked mannan is recognized by mannan receptor (MR), dectin-2, and Fc*γ*R [46, 47]. Phospholipomannan-(PLM-) coated *β*-glucans are recognized by both TLR-6 and TLR-2 [53, 54]. Other receptors may involve the signaling pathway of another PRR. For example, galectin-3, which recognizes *β*-mannosides, signals through TLR-2 (represented by a curved arrow) [47, 49] dectin-1, when activated by *β*-glucans can signal to activate the nuclear factor kappa-lightchain enhancer of activated B cells (NF-*κ*B) on its own or with the help of TLR-2 [4, 45]. Fc gamma receptor (Fc*γ*R) may signal through dectin-2 when activated by *N*-linked mannan [3, 45, 47, 55]. Recognition of these fungal cell wall components mediates fungal recognition and defense by the host. Recognition by host PRRs usually involves signaling through NF-*κ*B and activation of proinflammatory cytokines, such as TNF-*α*, or in some instances, and anti-inflammatory cytokines such as interleukin (IL)-10 [45]. The possibility of an alternative chitin receptor exists, activation of which leads to the recruitment of IL-4 producing cells [45, 59]. However, chitin has been shown to function as a T helper (Th)1 immune modulator, which stands in contrast an IL-4 associated Th2 response [60–64].

Generally, the innate immune system's response to PAMPs, which include glycosides, glycolipids, and carbohydrates, among others, involves pattern recognition receptors (PRRs) that are expressed by phagocytes. Pattern recognition then leads to a cascade of cellular signaling that activates phagocytes for defense [3, 26]. The recognition of fungal cellular features, in particular fungal cell wall components, by the immune system of the host is an important element for mounting an antifungal defense response [3, 5, 26, 43]. The fungal cell wall is composed of various mannoproteins, *β*-glucans as well as a thin, rigid layer of chitin (Figure 1). Many PRRs interact with fungal cell wall components. For example, mannoproteins with *O*-linked protein-carbohydrate conjugations are recognized by toll-like receptor (TLR)-4 [3, 44, 45], while mannoproteins that are N-linked can be recognized by dectin-2, mannose, and Fc*γ* receptors [3, 45–47] (Figure 2). The galectin-3 receptor recognizes *β*-mannosides [44, 48–50]. *β*-glucan is recognized as a PAMP by dectin-1 [4, 6] and when coated by phospholipomannan it is also recognized by both TLR-6 and TLR-2 [51–54]. Complement-coated *β*-glucan is recognized by complement receptor-3 [55, 56]. Dectin-1 recognition of *β*-glucans results in an MYD88-dependent pathway activation [3, 5, 44, 55, 57]. And finally, fungal CpG DNA is recognized by the intracellular receptor TLR-9 [58] (Figure 2). Recognition of fungal cell wall components by these PRRs generally leads to the nuclear factor kappa-lightchain enhancer of activated B cells (NF-*κ*B) signaling; this results in the activation of proinflammatory cytokines, such as tumor necrosis factor (TNF)-*α*, or anti-inflammatory cytokines, such as interleukin (IL)-10 (Figure 2). Whether chitin in the fungal cell wall is recognized as a PAMP, and if a specific chitin receptor exists as a PRR, remains unknown; yet it is a very likely possibility [3, 7].

## 4. Chitin as an Immune Modulator

Intranasal or intraperitoneal chitin administration to mice caused an immunological preactivation effect, called priming, in alveolar macrophages and natural killer (NK) cells [65]. Shibata et al. examined the effects of chitin particle sizes on cellular responses, in particular macrophage activation and priming. Balb/c mouse splenocytes that were cocultured with chitin particles (50–100 *μ*m), produced, and secreted IL-12, TNF-*α*, and IFN-*γ* [60]. However, intravenous injection of phagocytosable small chitin particles (1–10 *μ*m) into C57 mice resulted in a macrophage priming that was dose dependent [61]. When utilizing a SCID mouse model instead of the C57 mice, the same chitin-macrophage priming effect was also found. Because SCID mice lack mature B and T cells, the authors concluded that neither T nor B lymphocytes were required for chitin-induced macrophage priming. An NK cell depletion experiment with anti-NK1.1 antibodies (anti-CD161c) then demonstrated a requirement for NK cells and NK-secreted IFN-*γ* in chitin-induced macrophage priming [61]. However, as we describe below, chitin particles can also be used to activate macrophages and monocytes directly in cell-culture experiments.

It should be noted that chitin can also serve as an immunoadjuvant [65]. Orally administered chitin suppressed the production of T helper (Th)2 cytokines and immunoglobulin (Ig)E in a ragweed allergy mouse model and induced IFN-*γ* instead [62]. In addition, when used as an adjuvant, chitin produced Th1 responses comparable to other adjuvants, including heat-killed *Mycobacterium bovis*, Freud's complete adjuvant, and the Bacillus Calmette-Guérin vaccine [63]. Chitin produced effects similar to those of a Th1-promoting adjuvant in mouse models of ovalbumin-induced asthma and allergic hypersensitivities induced by the house dust mite *Dermatophagoides pteronyssinus* and by the fungal pathogen *A. fumigatus *[64, 66]. Chitin administration significantly reduced allergen-induced serum IgE levels and lung inflammation. Th1 cytokines IL-12, IFN-*γ*, and TNF-*α* were elevated, while IL-4 levels were decreased in mice-administered chitin as compared to controls [64, 66]. These and other studies strongly suggest that the immune system possesses a chitin recognition mechanism.

## 5. Mammalian Chitinases

Another immune response that may correlate with chitin recognition is the production of chitin-degrading enzymes, known as chitinases, by humans and other mammals. Chitinases belong to the glycosyl hydrolase 18 family, which is comprised of various proteins found in a wide range of organisms, including plants, bacteria, fungi, insects, protozoa, and mammals [13]. Six proteins with homology to chitinases have been identified in mammals. These include CHIT-1 and AMCase, which are the only two enzymatically active human chitinases able to hydrolyze chitin [11, 12, 14]. The other four of these highly homologous members of the chitinase family contain amino acid substitutions at their active sites, rendering these proteins noncatalytic. These noncatalytic chitinases are referred to as chi-lectins or chitinase-like proteins, and include chitinase-3-like protein 1 (CHI3L1, also known as YKL-40, Hcgp39, or GP39), stabilin-interacting chitinase-like protein (SI-CLP), YKL-39 (chitinase 3-like protein 2), and oviductin [13].

CHIT-1 is highly expressed by activated macrophages and is used as a marker for macrophage stimulation, suggesting a possible role in innate immunity [67, 68]. It was first discovered in the plasma of patients with Gaucher's disease; a disease characterized by the accumulation of lipid-laden macrophages [68–70]. The use of a chitinase detection assay, which measures the presence of chitinase activity via cleavage of the fluorogenic substrate 4-methylumbelliferyl chitotriosidase, showed that CHIT-1 levels were elevated several hundred-fold in the plasma of patients with Gaucher's disease. Therefore, CHIT-1 is now being used as a biomarker for the diagnosis of Gaucher's disease [68, 69, 71]. These findings drew attention to the cloning and further characterization of CHIT-1 [72, 73] and the discovery of the other enzymatically active human chitinase, AMCase. The sequence of AMCase is highly homologous to that of CHIT-1; however, AMCase is unique in that it functions strongest in acidic pH environments. Consistently, it was first found highly expressed in the stomach, intestinal tissue, and more recently is being studied as a biomarker for asthma and other hypersensitivities [11, 12, 27, 74].

Evolutionarily, chitinase production plays an important role in the life cycles of chitin-containing organisms such as fungi, insects and crustaceans, in which it is involved in either cell wall remodeling or molting. However, because mammals do not produce chitin, the physiological function of these chitinases and chi-lectins remains unclear, but various studies suggest that their function may lie in the digestion of chitin-containing foods and defense against chitin-containing pathogens and parasites [11, 13, 75].

## 6. Chitinases in Experimental Antifungal Therapy

Chitinases have also been investigated for their potential use in antifungal therapy. Low concentrations of recombinant human CHIT-1 degraded the cell walls of *Cryptococcus neoformans* and visibly inhibited its growth *in vitro *[67]. Morphological changes, such as atypical blebs, hyphal tip bursting, and restrictions of hyphal growth, were also observed for *Mucor rouxii* and *C. albicans* in the presence of recombinant CHIT-1 [67]. In addition, recombinant human CHIT-1 induced a dose-dependent improvement in the survival of mice with *C. albicans* and *A. fumigatus* infections [67]. Recently, it was shown that the culture medium conditioned by Chinese hamster ovary cells that had been retrovirally transfected with the human *CHIT-1* gene had antifungal activity [25]. These modified Chinese hamster ovary cells were then encapsulated in alginate microspheres and injected subcutaneously into BALB/c mice, where they continuously secreted active CHIT-1, and after infection with *C. neoformans,* mice harboring these cells had significantly lower fungal burden [25]. Therefore, the authors suggested that a continuous supply of active CHIT-1 should be explored in future gene therapies to prevent fungal infections.

## 7. Mammalian Chitinase Responses to Inflammation and Fungal Infections

Multiple stimuli, such as exposure to prolactin, interferon gamma (IFN-*γ*), lipopolysaccharides (LPS), and TNF-*α* can upregulate chitinase activity in human monocytes and macrophages, indicating a possible role for chitinase activity in inflammation [16–19]. Chitinase activity was reported to be upregulated as a result of various diseases, including candidiasis [76], *Wuchereria bancrofti *infections (filariasis) [21], and helminth infections [77, 78]. AMCase activity is highly upregulated in individuals suffering from asthma, chronic rhinosinusitis, or allergic bronchopulmonary aspergillosis [77, 78]. In addition, chitinase activity has been linked to fungal infections. In 1996, Overdijk et al. showed that, in guinea pigs, chitinase activity was induced after systemic infection with *A. fumigatus *[15, 79]. Furthermore, mice with pulmonary *C. neoformans *exposure had increased AMCase chitinase activities in the airways [80]. Intraperitoneal injections of zymosan, a yeast-cell wall-derived product that contains beta-glucans and small quantities of chitin, was shown to increase serum chitinase activity of rats [81].

Although chitinase activity does not appear to be specific for fungal infections, as it is also upregulated in other diseases, there appears to be a correlation between chitinase activity and inflammation as well as with disease induced by chitin-containing pathogens. These findings suggest that mammalian chitinase responses to fungal infections and other parasitic infections may be triggered by the host response to a chitin-containing pathogen.

## 8. Chitinase Induction and Regulation

Little is known about how host chitinase activity is induced, but there is some indication that chitinase production and chitin recognition could be linked. Gorzelanny et al. used MALDI-TOF mass spectrometry to analyze the degradation of chitin by chitotriosidase and followed the stimulation of human monocyte/macrophage with a chitin hexamer [82]. These studies revealed that chitinases degrade chitin into smaller chitin-oligomers that in turn enhance the stimulation of macrophages, leading to more chitinase production [82]. However, the feedback mechanism of chitin recognition and chitinase secretion suggested by this study is still unclear and the signaling pathways involved are not fully understood.

Other chitin stimulation experiments revealed some aspects of the mechanism involved in the recognition of chitin and chitin-containing parasites by immune cells. Jumonji domain containing-3 (Jmjd3), a histone 3 Lys27 (H3K27) demethylase, along with Irf4 transcription factor, was determined to be essential for macrophage colony-stimulating factor (M-CSF)-bone-marrow-derived M2 macrophage polarization in response to *Nippostrongylus brasiliensis* helminth infection and chitin inoculation [59]. Another group found that mice exposed to *N. brasiliensis* helminth infection showed tissue invasion by macrophages and IL-4- and IL-13-producing immune cells as well as eosinophil recruitment [83]. Furthermore, transgenic mice that overexpressed *AMCase* in the lung, and were also exposed to *N. brasiliensis,* showed diminished infiltration of immune cells. The diminished infiltration of cells was likely due to *N. brasiliensis* chitin degradation and removal [83]. The same effect was observed when chitin alone was injected, and the effect was sustained, even in TLR-4-deficient animals [83]. The latter effect is interesting, because TLR-4, which recognizes LPS and leads to activation of the innate immune system, was previously considered a possible chitin PRR candidate [44]. The observed recruitment of IL-4 producing immune cells by chitin stands in stark contrast to the previously observed Th1 immune response induced by chitin when used as an adjuvant (see above). IL-4 is a typical Th2 response-inducing cytokine. It is possible though, that the IL-4 production by recruited immune cells is a secondary effect that requires other chemokines or other chemoattractants to be produced by primary chitin-sensing cells.

In contrast to TLR-4, TLR-2 and the IL17A receptor (IL-17AR) may at least be partially involved in chitin recognition. Da Silva et al. reported that mouse macrophages stimulated with chitin particles had increased levels of IL-17 protein and IL-17AR mRNA, and the increase in IL-17 was mediated via the TLR-2 pathway. *In vivo* investigations demonstrated that chitin induces acute pulmonary inflammation in wild-type mice, but not in TLR-2 knockout mice [84]. Therefore, it is possible that TLR-2 and IL-17AR are somehow involved in the recognition of chitin. TLR-2 is known to recognize bacterial particles, LPS, and more interestingly, zymosan, which contains chitin (see above) [44].

**Figure 3:**
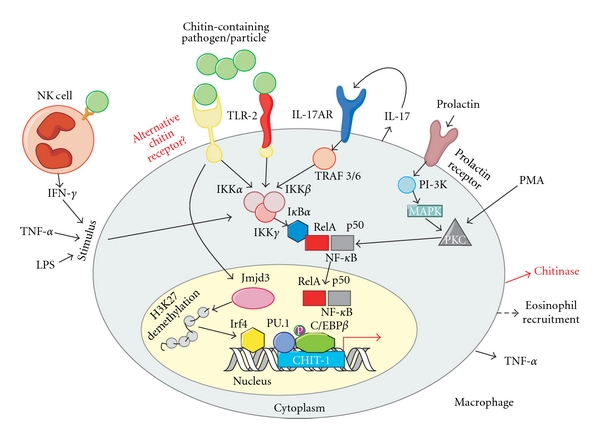
Hypothetical model for a molecular chitinase response to chitin-containing pathogens or particles. Chitin recognition leads to the expression of chitinase, TNF-*α*, IL-17, and eosinophil recruitment [83, 84]. NF-*κ*B serves as the primary signaling pathway involved in the recognition of most fungal cell wall components (see Figure 2) [45]; therefore, its involvement in the expression of chitinase in response to a chitin-containing pathogen or particle is highly likely. Secretion of IFN-*γ* by NK cells induces macrophage priming caused by stimulation with a chitin-containing pathogen or particle [60–63]. IFN-*γ*, LPS, TNF-*α*, and PMA up-regulate chitinase activity [16–19], possibly through an NF-*κ*B inflammatory-mediated pathway. IL-17 secretion upon macrophage stimulation with chitin increased levels of IL-17-AR and was TLR-2 dependent [84]. Either TLR-2 or an alternative chitin-specific receptor may recognize chitin-containing pathogens or particles and mediate chitinase activity. Prolactin stimulation of macrophages leads to chitinase expression via a PI-3K, MAPK, and NF-*κ*B pathway [16–19]. Activation of Jmjd3, leads to demethylation of H3K27 and recruitment of the transcription factor Irf4, which is associated with M2 macrophage polarization in response to chitin stimulation [59]. Expression of the chitinase encoded by the CHIT-1 gene is regulated via PU.1 and C/EBP*β*. The latter is phosphorylated (p) to induce CHIT-1 expression [85]. NF-*κ*B, nuclear factor kappa-light-chain-enhancer of activated B cells; IFN-*γ*: interferon gamma; NK cells: natural killer cells; LPS: lipopolysaccharide; TNF-*α*: tumor necrosis factor alpha; PMA: phorbol 12-myristate 13-acetate; IL-17: interleukin-17; IL-17-AR: interleukin-17A receptor; TLR-2: toll-like receptor-2; PI-3K: phosphatidylinositol 3-kinase; MAPK: Mitogen-activated protein kinase; Jmjd3: Jumonji domain containing-3; H3K27: histone 3 Lys27 demethylase; CHIT-1: chitotriosidase; C/EBP*β*: CCAAT-enhancer-binding protein beta.

Portions of the downstream signaling pathway that leads to chitinase expression have been analyzed. *CHIT-1* mRNA expression in monocytes increases upon treatment with phorbol 12-myristate 13-acetate (PMA), which induces differentiation of monocytes into activated macrophages [85]. In addition, *CHIT-1* gene activation is accompanied by the binding of phosphorylated CCAAT-enhancer-binding protein (C/EBP)*β* and the transcription factor PU.1 to the promoter region of CHIT-1 (Figure 3) [85]. The upstream molecular signaling pathway leading to *CHIT-1 *gene activation and chitinase induction has not been determined; however, roles for some key proteins involved in chitinase regulation have been noted [16–19]. Chitinase gene expression and activity was induced by human-monocyte-derived macrophages after prolactin stimulation in both a dose- and time-dependent manner [17]. Because prolactin has similar structural properties as some proinflammatory cytokines, alternative stimulations were preformed with IFN-*γ*, TNF-*α*, and LPS, and, as a negative control, with IL-10, which has anti-inflammatory properties. Chitinase activity was elevated in human monocyte-derived macrophages after stimulation with IFN-*γ*, TNF-*α*, and LPS and was significantly decreased after stimulation with IL-10 [16, 19]. These findings may indicate the involvement of chitinase activity induction during inflammatory conditions. Prolactin stimulation of human monocyte-derived macrophages was also performed in the presence or absence of specific kinase inhibitors [18]. The phosphatidylinositol 3-kinase (PI3-K) inhibitors wortmannin and LY-294002 reduced chitinase activity, as did the protein tyrosine kinase inhibitor genistein, the mitogen-activated kinase (MAPK) p38 inhibitor SB203580, and the MAPK p44/42 inhibitor U0126. No effect was observed on prolactin-mediated chitinase induction when the controversial protein kinase C inhibitor rottlerin was used, nor was an effect seen with PP2, a Src inhibitor, or AG490, a JAK2 inhibitor [18]. Accordingly, CHIT-1 induction can be mediated via a PI3-K/MAPK pathway (Figure 3).

## 9. Polymorphisms in Chitinase Proteins

The induction of chitinase activity as an immune response to various stimuli such TNF-*α*, prolactin, and chitin, and in response to fungal infections suggests that chitinases are indeed involved in the host's immune response to a pathogenic fungal invader. However, multiple known polymorphisms can affect chitinase activity, the most prominent being a 24-bp duplication in the *CHIT-1* gene. The *CHIT-1* gene is composed of 12 exons and the protein is secreted as two isoforms. The major isoform has a molecular mass of 50 kDa, undergoes posttranslational modifications, including *O*-linked glycosylation of the C-terminal region (which contains the chitin-binding domain) and is alternatively spliced into the 39 kDa minor isoform [20, 68]. Sometimes, a 24-bp duplication occurs in exon 10 of *CHIT-1* that causes a downstream cryptic 3′ splice site that generates mRNA with an in-frame deletion of 87 nucleotides (29 amino acids). This mutant protein can bind chitin particles, but cannot degrade chitin [20, 86]. Macrophages from individuals with this 24-bp duplication in the *CHIT-1* gene produced *CHIT-1* RNA and small amounts of a 47 kDa protein, but no enzymatically active chitotriosidase [20]. Approximately 30–40% of the human population is heterozygous and 3–6% is homozygous for this duplication [20, 77, 86]. The use of chitinase activity as a disease biomarker may therefore be limited to patients with at least one wild-type *CHIT-1* allele.

The effect of environmental conditions on the occurrence of the most prominent chitinase polymorphism, the 24-bp duplication, was studied by Malaguarnera et al. DNA analysis was performed to compare the frequency of the exon 10 duplication allele in individuals from Mediterranean countries and sub-Saharan regions. This study found a higher frequency of individuals homozygous for the 24-bp duplication in Sicily and Sardinia, 3.73% and 5.45%, respectively, than in people from Benin and Burkina Faso (frequency of 0% homozygous for the duplication). The authors concluded that the presence of the *CHIT-1*-inactivating 24-bp duplication in Sicily and Sardinia was due to the improved, more sanitary environmental conditions as compared to Benin and Burkina Faso, which still face widespread parasitic diseases and the presence of multiple chitin-containing pathogens [77]. The lack of chitotriosidase activity in people with these polymorphisms may be compensated for by AMCase chitinase activity. However, there are also various polymorphisms that affect AMCase activity [22, 23, 87]. Therefore, a thorough immunogenetic haplotype analysis that involves *CHIT-1* and *AMCase* alleles, as well as chitin sensing and chitinase regulation pathways is needed to investigate the significance of human chitinase responses to fungal infections. It is possible that the dysregulation of chitin sensing or chitinase induction pathways could be associated with altered susceptibility for IFIs.

## 10. Concluding Remarks and Future Directions

An efficient method for early diagnosis and treatment of IFIs is needed [27, 30]. Exploiting host responses to IFIs could help to better understand fungal recognition by the immune system, and may reveal potential diagnostic markers of IFIs. A substantial increase in chitinase activity, in conjunction with other IFIs clinical signs and symptoms, or in conjunction with the *β*-1,3-glucan assay could be a biomarker indicative of a beginning fungal infection. Chitinase activity appears to play an important role in various diseases [13, 68], and therefore, a clear understanding of the processes of chitinase induction and regulation is desirable.

Chitinases can be induced by various stimuli including prolactin, TNF-*α*, IFN-*γ*, and PMA. And recombinant human CHIT-1 has demonstrated antifungal properties both *in vivo* and *in vitro *[25, 67]. Therefore, it is conceivable that artificial induction of chitinase production in patients that are at risk of fungal infections could increase their resistance to fungal pathogens. This strategy would be most effective in patients with genes encoding catalytically active chitinases. In summary, chitinase-based diagnostic assay or antifungal therapeutics may be developed in the near future.
